# Decoding PFAS immunotoxicity: a NAMs-based comparison of short vs. long chains

**DOI:** 10.3389/ftox.2025.1665163

**Published:** 2025-11-19

**Authors:** Martina Iulini, Valentina Galbiati, Marina Marinovich, Emanuela Corsini

**Affiliations:** Laboratory of Toxicology and Risk Assessment, Department of Pharmacological and Biomolecular Sciences “Rodolfo Paoletti”, University of Milan, Milan, Italy

**Keywords:** new approach methodologies (NAMs), PFAS, hazard identification, human PBMCs, dendritic cells, *in vitro*

## Abstract

**Introduction:**

Per- and polyfluoroalkyl substances (PFAS) are persistent environmental pollutants with potential immunotoxic effects. Most toxicological studies have focused on long-chain PFAS such as perfluorooctanesulfonic acid (PFOS) and perfluorooctanoic acid (PFOA). However, short-chain and ultra-short-chain alternatives, including trifluoroacetic acid (TFA), are increasingly used despite limited toxicological data.

**Methods:**

This study evaluated and compared the immunotoxic effects of PFAS with varying chain lengths—long-, short-, ultra-short-chain compounds, and fluoropolymer representatives (polytetrafluoroethylene, PTFE)—using human-relevant new approach methodologies (NAMs). Two complementary in vitro models were employed. Peripheral blood mononuclear cells (PBMCs) from healthy donors to assess antibody (IgG and IgM) production. THP-1-derived dendritic cells (DCs) to evaluate maturation marker expression (CD83, CD86, HLA-DR). Environmentally and occupationally relevant PFAS concentrations were tested.

**Results:**

PFOS, PFOA, and perfluorononanoic acid (PFNA) suppressed antibody production and impaired DC maturation in a concentration-dependent manner, consistent with previous in vivo and epidemiological data. Short-chain PFAS (PFHxS, PFBS, PFHxA, PFBA) showed modest to intermediate immunomodulatory activity, with subtle immunosuppressive trends in female donors. Notably, TFA reduced antibody production at levels comparable to PFOS, indicating that chain length alone is not a reliable predictor of immunotoxic potential. PTFE exhibited no suppressive effects; instead, increased antibody release was observed in female donors, suggesting possible sex-dependent immunostimulation.

**Discussion:**

Findings support a nuanced, compound-specific approach to PFAS risk assessment rather than a simple long- vs. short-chain distinction. In vitro NAMs provided mechanistic, human-relevant insights and reinforce their integration into regulatory frameworks.

## 1 Introduction

Per- and polyfluoroalkyl substances (PFAS) are a large class of synthetic compounds with exceptional chemical stability but also environmental persistence and bioaccumulative potential (Buck et al., 2011). PFAS are widely used in industrial and consumer products due to their unique surfactant properties, stability, and resistance to heat and stains. The most studied PFAS are perfluorooctanoic acid (PFOA), perfluorooctane sulfonate (PFOS), perfluorononanoic acid (PFNA) and perfluorohexane sulfonic acid (PFHxS). They have historically been employed in applications including firefighting foams, stain-resistant textiles, and non-stick cookware coatings. These substances account for the majority of PFAS detected in human blood (Domingo and Nadal, 2019; EFSA Panel on Contaminants in the Food Chain et al., 2020). PFAS are classified as sulfonic (PFSAs) or carboxylic (PFCAs) acids based on the functional group attached to the perfluorinated carbon chain. Sulfonic acids contain a -SO_3_H group, while carboxylic acids contain a -COOH group. They are further categorized by chain length: long-chain PFCAs have the formula C_n_F_2n+1_COOH with n ≥ seven (e.g., PFOA), while long-chain PFSAs have C_n_F_2n+1_SO_3_H with n ≥ six (e.g., PFOS); those with fewer carbon atoms are referred to as short-chain PFAS, such as perfluorobutanesulfonic acid (PFBS) and perfluorohexanoic acid (PFHxA) (Buck et al., 2011; Wang et al., 2013). Short-chain PFAS have more recently been introduced as replacements for many PFAS applications, driven by regulatory restrictions on long-chain compounds (ECHA, 2023). Long-chain PFAS are favored for applications requiring strong surface activity and durability, while short-chain PFAS are preferred for their lower bioaccumulation potential in human; however, they have a higher environmental mobility (Gomis et al., 2018; Wang et al., 2013).

The pharmacokinetic behavior of PFAS varies considerably depending on their carbon chain length. This has a significant impact on their bioaccumulation and persistence in the human body. Long-chain PFAS bind strongly to serum proteins such as albumin, resulting in prolonged half-lives in humans, ranging from several years for PFOS and PFOA (respectively 4.8 and 3.5 years), and accumulation in protein-rich organs like the liver, kidneys, and spleen (Han et al., 2003; Wang et al., 2025). A key factor contributing to the extended half-life of long-chain PFAS is their efficient renal tubular reabsorption. These compounds are actively reabsorbed in the kidneys via organic anion transporters, reducing their urinary excretion and leading to bioaccumulation (Niu et al., 2023). This mechanism can result in persistent systemic exposure, even after environmental or occupational sources have been eliminated. In contrast, short-chain PFAS generally have much shorter biological half-lives, which are often measured in days or months rather than years (Schrenk et al., 2020; Domingo, 2025). Their weaker binding to serum proteins and reduced affinity for renal reabsorption transporters facilitate their elimination via urine more rapidly. Consequently, short-chain PFAS tend to be less bioaccumulative in humans and animals. However, their increased environmental mobility and persistence still pose significant health risks (Gomis et al., 2018). Despite their shorter half-lives, these compounds can accumulate in tissues related to the immune system, such as the spleen, due to their physicochemical properties and exposure patterns (Nielsen et al., 2024; Domingo, 2025). These differences in toxicokinetic underpin variations in systemic exposure and potential health effects. While long-chain PFAS are facing global restrictions due to their persistence and toxicity, short-chain alternatives have been marketed as safer. It has become clear that more recent alternatives are equally or even more toxic than conventional PFAS, causing liver damage, thyroid dysfunction, neurotoxicity, and reproductive toxicity (Gaballah et al., 2020; Zhang et al., 2021; Feng et al., 2024). Beyond chain length, other categories of PFAS are increasingly recognized for their potential toxicological relevance. These include branched isomers, fluorotelomers, and ether-based PFAS such as hexafluoropropylene oxide dimer acid (HFPO-DA, commonly known as GenX). Emerging evidence suggests that these compounds may exert immunotoxic, hepatotoxic, and endocrine effects through mechanisms not strictly related to carbon chain length, underscoring the need for compound-specific assessment (Wang et al., 2013; Gebbink et al., 2017).

Immunotoxicity is a well-recognized adverse outcome of PFAS exposure (Schrenk et al., 2020; EPA, 2016). Animal studies have repeatedly shown that exposure to PFOS, PFOA and related compounds suppresses antibody production, impairs T-cell function and alters cytokine profiles (Peden-Adams et al., 2008; DeWitt et al., 2012; Dong et al., 2021). Epidemiological evidence corroborates these findings, linking elevated serum PFAS concentrations with reduced vaccine antibody titres particularly in children (Grandjean et al., 2012; Abraham et al., 2020; Zhang et al., 2022). Higher PFOS levels have been correlated with reductions of 30%–50% in tetanus/diphtheria vaccine titers, particularly in children (Grandjean et al., 2012). Emerging evidence suggests that exposure to PFAS may impair vaccine-induced immunity also in adults. For instance, a prospective study of adults who were vaccinated against influenza found an inverse relationship between serum PFOA levels and antibody concentrations (Looker et al., 2014). Other studies have reported similar associations between PFAS exposure and reduced antibody responses to rubella in adults, especially a significant association between rubella titres and PFOA was found in men but not women (Stein et al., 2016; Pilkerton et al., 2018). Furthermore, PFAS exposure has been linked to an increased severity the disease caused by the SARS-CoV-2 virus (Grandjean et al., 2020; Catelan et al., 2021) as well as a higher risk of other infectious diseases (Timmermann et al., 2020; Bulka et al., 2021). Studies of mother–child cohorts have also reported greater susceptibility to infections related to prenatal PFAS exposure (Granum et al., 2013; Ait Bamai et al., 2020; Dalsager et al., 2021; Wang et al., 2022). Additionally, PFAS exposure has been positively associated with asthma development (Dong et al., 2013; Timmermann et al., 2017; Averina et al., 2019). Studies have also shown positive and negative associations between PFAS exposure and allergic outcomes (Ait Bamai et al., 2020; Averina et al., 2019). Finally, autoimmune diseases associated with PFAS exposure have been documented in populations exposed to extremely high environmental concentrations (Steenland et al., 2015). Although the findings vary somewhat due to differences in exposure profiles and the timing of assessments, the overall evidence suggests that PFAS-induced immunosuppression is not confined to paediatric populations. Moreover, recent evaluations by the International Agency for Research on Cancer (IARC) have classified PFOA as “carcinogenic to humans” (Group 1) and PFOS as “possibly carcinogenic to humans” (Group 2B), recognizing their carcinogenic potential alongside their immunotoxic effects (International Agency for Research on Cancer, 2023). Although short-chain PFAS have generally been considered less immunotoxic, recent studies suggest that they may induce oxidative stress and mitochondrial dysfunction in immune cells (Liu et al., 2023; Obiako et al., 2024). *In vitro*, the surveys reveal that PFBS and PFHxA induce reactive oxygen species (ROS) in THP-1 macrophages at environmentally relevant concentrations (≥1 μM) independently of Peroxisome Proliferator-Activated Receptor alfa (PPAR-α) activation (Amstutz et al., 2024). This oxidative burden impairs ATP synthesis, triggers apoptosis in developing B cells and disrupts neutrophil chemotaxis (Liu et al., 2023; Amstutz et al., 2024; Obiako et al., 2024). *In vivo* rodent experiments suggest that short-chain PFAS do not significantly suppress the T-dependent antibody response (TDAR) at doses similar to those of long-chain (Hughes et al., 2025). However, subtle alterations in immune cell populations have been observed, including reduced numbers of B lymphocytes and natural killer cells (Woodlief et al., 2021). Similarly, studies on zebrafish have demonstrated that short-chain PFAS, such as perfluorobutanoic acid (PFBA), can impair antibacterial defense mechanisms and modulate innate immune pathways involving Toll-like receptor signalling and MyD88 activation. However, the effects appear to be less pronounced compared to those of longer-chain homologues (Tian et al., 2023). Taken together, these findings suggest that short-chain PFAS are not immunologically inert and may disrupt immune system homeostasis, albeit at higher exposure levels. However, the immunotoxic potential of many short-chain PFAS remains poorly characterized.

While traditional animal models are informative, they often lack the human relevance and mechanistic resolution required for modern risk assessment. Differences in immune system development, receptor expression and kinetic between humans and animals can restrict the application of results. Consequently, the scientific community and regulatory agencies are increasingly adopting new approach methodologies (NAMs), such as human cell-based *in vitro* models, computational simulations and omics-based approaches, to better elucidate chemical immunotoxicity and facilitate the shift towards non-animal testing strategies (Fenton et al., 2021). *In vitro* methods allow us to replicate key human immune endpoints including antibody production and cytokine signaling and provide mechanistic insights. For instance, Namalwa B cells and human peripheral blood mononuclear cells (PBMCs) have been employed to evaluate the inhibitory effects of PFAS on antigen-specific antibody production (Janssen et al., 2024; Corsini et al., 2024). These models provide a physiologically relevant context, enabling the evaluation of cellular interactions and signaling pathways that are critical for immune responses. Using PBMC, we demonstrated that PFOS is significantly more potent *in vitro* than PFOA in suppressing antibody responses (Corsini et al., 2024). These results are consistent with epidemiological data indicating that populations with higher PFOS exposure exhibit greater reductions in vaccine antibody titers (Grandjean et al., 2012; Zhang et al., 2022). Physiologically based kinetic (PBK) modelling can be integrated with *in vitro* toxicity data and human absorption, distribution, metabolism and excretion profiles in order to predict tissue-specific concentrations of PFAS. This approach is particularly valuable for translating *in vitro* findings into *in vivo* risk assessments. PBK models have been used to simulate exposure to long-chain PFAS and predict its accumulation in key immune tissues (Loccisano et al., 2011; Corsini et al., 2024). The results of these simulations have shown a strong correlation with observed splenic PFOS concentrations in cohorts with high exposure, providing mechanistic support for the biological plausibility of the immunosuppressive effects observed *in vitro* and in epidemiological studies (Deepika et al., 2021). Initiatives such as the Tox21 program have screened thousands of chemicals, including PFAS, for their effects on various biological endpoints. By analyzing these data, researchers have identified the inhibition of NF-κB signaling as a shared endpoint for both long-chain and short-chain PFAS (Ooka et al., 2024). NF-κB is a critical transcription factor involved in immune cell activation and cytokine production. Inhibition of NF-κB by PFAS can lead to reduced immune responses, which is consistent with the immunosuppressive effects observed *in vitro* and *in vivo* studies (Ehrlich et al., 2023).

In this context, the present study employs NAMs to directly compare the immunotoxic effects of representative long- (PFOA, PFOS, PFNAX) and short-chain (PFHxS, PFBS, PFHxA and PFBA) PFAS on the human immune system. By directly comparing these effects, we aimed to establish whether short-chain alternatives, introduced as replacements for the now well-known and regulated long-chain PFAS, are safer or whether they pose similar risks. In addition, we also tested the effect on immunoglobulin production of two additional PFAS at opposite ends of the carbon-fluorine chain spectrum: the ultra-short-chain PFCAs compound trifluoroacetic acid (TFA) and the fluoro polymer polytetrafluoroethylene (PTFE). TFA is currently considered the most abundant PFAS in the global environment. It is a terminal degradation product of several fluorinated compounds and has been detected in multiple environmental matrices including drinking water, rainwater, and agricultural products (Arp et al., 2024). Elevated environmental concentrations of TFA arise not only from its direct use, but also from the degradation of various precursors, including refrigerant chemicals, agrochemicals, pharmaceuticals, and fluoropolymers (Arp et al., 2024). Despite its short chain and low bioaccumulation potential, its high environmental persistence and potential for chronic exposure have raised concerns regarding possible immunotoxic effects, which remain largely unexplored. On the other hand, PTFE is a high-molecular-weight fluoropolymer widely used in industrial and consumer applications due to its remarkable thermal and chemical resistance. PTFE is generally regarded as biologically inert and non-bioavailable under standard use conditions. However, its production may involve or release smaller PFAS molecules, including PFOA, especially in poorly controlled manufacturing settings. Including PTFE in our analysis allowed us to verify its inertness in a human-relevant immunotoxicity model and to explore whether it may exert any previously unrecognized immunomodulatory effects.

Specifically, as *in vivo* the decrease in antibody production was identified as the critical endpoint, we investigated the effects on humoral immunity using PBMCs from healthy donors by assessing antibody production. In addition, we also investigated the effects of the short and long-PFAS on dendritic cell (DCs) activation and maturation. As an experimental model, THP-1 cell line was used. The use of these models enabled a robust comparison between long- and short-chain PFAS, providing important insights into their relative immunotoxic potential and supporting the identification of safer alternatives.

## 2 Materials and methods

Chemicals: PFOA (CAS #335-67-1, purity 95%), PFOS (CAS #1763-23-1, acid solution ∼40% in H_2_O), PFNA (CAS #375-95-1, purity 97%), PFHxS (CAS #3871-99-6 purity ≥98%), PFHxA (CAS #307-24-4, purity ≥97%), PFBA (CAS #375-22-4, purity 98%), PFBS (CAS #375-73-5, purity 98%), PTFE (CAS #9002-84-0), TFA (CAS #76-05-1, purity ≥99%), dexamethasone (DEX, 9α-fluoro-16α-methylprednisolone, CAS #50-02-2, purity ≥98%) and rapamycin (CAS # 53123-88-9, purity ≥95%) were purchased from Sigma-Aldrich (St Louis, MO, United States). All the chemicals were diluted in dimethyl sulfoxide (DMSO, CAS #67-68-5). The final concentration of DMSO in cell culture was 0.1%. PTFE was tested as a commercial powder (mean particle size ∼200 μm). The powder was suspended in DMSO and heated at 120 °C for 120 min, with vortexing every 30 min to enhance dispersion. The suspension was diluted into cell culture medium immediately before use. Recombinant human interleukin-4 (rhIL-4) and recombinant human granulocyte-macrophage colony-stimulating factor (rhGM-CSF) were purchased from ImmunoTools GmbH (Friesoythe, Germany), recombinant human tumor necrosis factor-alpha (rhTNF-α) and ionomycin were purchased from Sigma-Aldrich, recombinant human nterleukin-2 (rhIL-2) was purchased from Miltenyi Biotec (Bergisch Gladbach, Germany), ODN 2006 (ODN 7909) was purchased from InvivoGen (San Diego, CA, United States). rhIL-4, rhGM-CSF, rhTNF-α, ionomycin, rhIL-2 and ODN2006 were all diluted in Dulbecco’s phosphate-buffered saline (PBS).

### 2.1 Protocol for assessing the effect of PFAS on antibody release

PBMCs were isolated from anonymous buffy coats obtained from healthy male and female donors, which were purchased from Niguarda Hospital in Milan, Italy. The PBMCs were isolated using Ficoll gradient centrifugation and subsequently washed five times with PBS. For the experiments, the PBMCs were suspended in RPMI 1640 medium without phenol red, containing 2 mM L-glutamine, 100 IU/mL penicillin, 0.1 mg/mL streptomycin, 10 μg/mL gentamicin and 50 µM 2-mercaptoethanol. This was supplemented with 5% heat-inactivated human serum to create the complete medium. The cell culture medium, serum and all supplements were purchased from Sigma-Aldrich.

PBMCs (1.26 × 10^6^ cells/mL) were seeded in 48-well plates in complete medium. The cells were then treated with increasing non-cytotoxic concentrations of the selected PFAS (0.001, 0.1 and 10 μg/mL), rapamycin at a concentration of 2 ng/mL, or DMSO as the vehicle control. The cells were incubated at 37 °C in 5% CO_2_ for 24 h. The PBMCs were then stimulated with 1 μg/mL of ODN2006 and 100 IU/mL of rhIL-2 for 6 days.

To determine the release of total IgG and IgM after 6 days, PBMCs were harvested and centrifuged for 5 min at 2000 rpm and 25 °C. The resulting supernatant was collected and stored at −20 °C until measurement. The release of IgM and IgG, or total Ig (IgG + IgM), was evaluated using a custom ELISA assembled in-house from reagents purchased from Sigma-Aldrich. Briefly, 100 µL of anti-human IgG (Cat. No. I1886) and/or anti-human IgM (Cat. No. I0104) solutions at 1 μg/mL in PBS were plated in a 96-well plate and incubated overnight at 4 °C. Then, 100 µL of standard (0–1,000 ng/mL) or diluted samples in reagent buffer (PBS +0.5% bovine serum albumin +0.05% Tween 20) were plated and incubated for 2 hours at room temperature. Then, 100 µL of anti-human polyvalent Igs (Cat. No. A3313), diluted 1:2000 in reagent buffer, were added to the plates and incubated for 1 hour at room temperature. Finally, 100 µL of Sigma 104 substrate diluted in AP buffer (Cat. No. A4955) was added to the plates. Absorbance was read at 415 nm. The data was analyzed using the integrated SoftMax Pro 7.1.2 software. The results are expressed as the fold change of chemical-treated cells versus vehicle-treated cells, from this point on, the term to use is ‘stimulation index’ (SI).

### 2.2 Protocol for assessing the effect of PFAS on antigen presenting cells (APCs)

THP-1 cells (1 × 10^5^ cells/mL) were cultured for 5 days in the presence of rhIL-4 (1500 U/mL) and rhGM-CSF (1500 U/mL) to induce the characteristics of immature DCs (iDCs), following the protocol by Berges et al. (2005). The cells were maintained in RPMI 1640 medium supplemented with 2 mM glutamine, 0.1 mg/mL streptomycin, 100 U/mL penicillin, 50 µM 2-mercaptoethanol and 10% heat-inactivated fetal bovine serum (FBS) at 37 °C in a 5% CO_2_ atmosphere.

On day five, the cells (1 × 10^6^ cells/mL) were treated for 24 h with the increasing concentration of the selected PFAS (0.001, 0.1 and 10 μg/mL), DEX as a positive control (150 μg/mL) and DMSO as a vehicle control. This was done using RPMI 1640 medium without phenol red, supplemented with 2 mM L-glutamine, 0.1 mg/mL streptomycin, 100 U/mL penicillin, 50 µM 2-mercaptoethanol and 5% heat-inactivated, delipidated FBS. After this, a maturation cocktail containing rhIL-4 (3,000 IU/mL), rhGM-CSF (1,500 IU/mL), rhTNF-α (2,000 IU/mL) and ionomycin (200 ng/mL) was added to induce the development of mature DCs (mDCs). After 24 and 72 h, expressions of the key cell surface markers CD83, CD86 and HLA-DR were assessed to evaluate the maturation process and determine whether PFAS could interfere with it.

Cell surface marker expressions were analyzed using flow cytometry. The cells were stained with FITC/PE-conjugated antibodies that target CD40, CD80, CD83, CD86 and HLA-DR (BD Pharmingen™, Milan, Italy; ImmunoTools GmbH), or with isotype control antibodies. This was done in accordance with the supplier’s instructions at 4 °C. Fluorescence intensity was measured using a NovoCyte 3,000 flow cytometer and the data were quantified using NovoCyte software. Changes in marker expression were reported as the Stimulation Index (SI), which was calculated based on the mean fluorescence intensity (MFI) values of the treated versus control mDCs.

A more accurate and detailed explanation of both the entire protocol can be found in the standard operating procedures section of Corsini et al. (2024).

### 2.3 Statistical analysis

Results are expressed as mean ± standard error of mean (SEM) of 10 independent donors. Statistical analysis was performed using GraphPad Prism version 10.2.3 (GraphPad Software, La Jolla, CA, United States). Significant differences were determined using paired or unpaired T-test, or analysis of variance (ANOVA), followed, when significant, by an appropriate *post hoc* test, as indicated in the Figure legends. Effects were designated as significant if the p value was ≤0.05.

## 3 Results

Initial experiments were carried out to define concentrations that would not cause overt cytotoxicity in immune cell cultures. Cytotoxicity was systematically evaluated in both concentration- and time-dependent manners by measuring LDH release using the CyQUANT™ LDH Cytotoxicity Assay Kit (Invitrogen™, Carlsbad, California). The highest concentration tested was 10 μg/mL; this upper limit was chosen to reflect realistic human exposure levels detected in blood or serum, rather than to reach cytotoxic thresholds. The concentration range included both levels in the ng/mL range, representative of the general population, and µg/mL, representative of occupational or highly exposed individuals (Schrenk et al., 2020). The selection of these concentrations was made in consultation with experts from an EFSA-sponsored project and in accordance with EFSA’s current recommendations for immunotoxicity testing (Corsini et al., 2024). All tested concentrations were confirmed to be non-cytotoxic in our models (data not shown), and the nominal concentration range selected was 0.001, 0.1, and 10 μg/mL for all the PFAS tested. For clarity, the conversion of the tested nominal concentrations (µg/mL) into the corresponding molar concentrations (µM), based on the molecular weight of each PFAS, is reported in Supplementary Table S1.

### 3.1 Effects of PFAS on T independent antibody production

Tables 1, 2 summarize the effects of the tested PFAS on IgG and IgM antibody production in human PBMCs obtained from male and female donors, respectively. The results reveal differences in immunomodulatory activity that are both compound- and gender-specific.

**TABLE 1:** Effect of PFAS on antibody production in male donors.

Male donors	IgG			IgM		
	0.001 μg/mL	0.1 μg/mL	10 μg/mL	0.001 μg/mL	0.1 μg/mL	10 μg/mL
PFNA	0.92 ± 0.07	0.78 ± 0.09	0.58 ± 0.09**	0.86 ± 0.06	0.71 ± 0.06*	0.65 ± 0.09**
PFOS	0.73 ± 0.13	0.61 ± 0.11**	0.48 ± 0.13**	0.85 ± 0.01	0.73 ± 0.10*	0.68 ± 0.07**
PFOA	0.73 ± 0.09	0.66 ± 0.10**	0.67 ± 0.14*	0.84 ± 0.08	0.84 ± 0.04	0.81 ± 0.08
PFHxS	1.01 ± 0.06	0.89 ± 0.03	0.61 ± 0.08**	0.86 ± 0.09	0.76 ± 0.08	0.73 ± 0.05*
PFHxA	0.95 ± 0.05	1.02 ± 0.08	0.99 ± 0.09	0.84 ± 0.06	0.81 ± 0.06	0.72 ± 0.10*
PFBS	0.98 ± 0.03	1.00 ± 0.02	0.98 ± 0.04	0.82 ± 0.12	0.76 ± 0.11	0.61 ± 0.10**
PFBA	0.90 ± 0.13	0.87 ± 0.16	0.86 ± 0.12	0.71 ± 0.09*	0.96 ± 0.15	0.70 ± 0.08*

Male PBMCs, were exposed to increasing concentrations of chemicals for 24 h, then stimulated with ODN2006 and rhIL-2, for 6 days. The results are expressed as the SI, of IgG or IgM compared to the negative control (DMSO, 0 μg/mL–value 1.00 ± 0.00). Each value represents the SI, mean ± SEM, with n = 5 male donors. Statistical analysis was performed by two-way ANOVA, followed by Dunnett’s test for PFAS, vs. DMSO (0 μg/mL). Results were considered significant if p ≤ 0.05, with *p < 0.05, **p < 0.01 vs. Ctrl.

**TABLE 2:** Effect of PFAS on antibody production in female donors.

Female donors	IgG			IgM		
	0.001 μg/mL	0.1 μg/mL	10 μg/mL	0.001 μg/mL	0.1 μg/mL	10 μg/mL
PFNA	0.78 ± 0.08	0.68 ± 0.10	0.56 ± 0.09*	0.78 ± 0.08	0.68 ± 0.10	0.56 ± 0.09*
PFOS	0.78 ± 0.06	0.80 ± 0.07	0.72 ± 0.10	0.60 ± 0.10*	0.50 ± 0.12**	0.51 ± 0.14**
PFOA	0.72 ± 0.10	0.88 ± 0.08	0.86 ± 0.03	0.60 ± 0.13*	0.55 ± 0.11*	0.51 ± 0.11**
PFHxS	0.87 ± 0.08	0.85 ± 0.05	0.73 ± 0.06	0.85 ± 0.09	0.80 ± 0.09	0.72 ± 0.06
PFHxA	1.00 ± 0.03	0.99 ± 0.05	0.99 ± 0.06	0.85 ± 0.10	0.75 ± 0.11	0.71 ± 0.14
PFBS	0.92 ± 0.06	0.97 ± 0.04	1.01 ± 0.04	0.83 ± 0.14	0.68 ± 0.14	0.76 ± 0.16
PFBA	1.02 ± 0.03	1.10 ± 0.06	0.95 ± 0.04	1.00 ± 0.11	0.87 ± 0.12	0.90 ± 0.09

Female PBMCs, were exposed to increasing concentrations of chemicals for 24 h, then stimulated with ODN2006 and rhIL-2, for 6 days. The results are expressed as the SI, of IgG or IgM compared to the negative control (DMSO, 0 µg/mL–value 1.00 ± 0.00). Each value represents the SI, mean ± SEM, with n = 5 female donors. Statistical analysis was performed by two-way ANOVA, followed by Dunnett’s test for PFAS, vs. DMSO (0 μg/mL). Results were considered significant if p ≤ 0.05, with *p < 0.05, **p < 0.01 vs. Ctrl.

In male donors (Table 1), long-chain PFAS such as PFNA, PFOS and PFOA consistently suppressed IgG production in a concentration-dependent manner. The most pronounced reductions were observed at the highest tested concentration (10 μg/mL). For instance, PFNA reduced IgG SI to 0.58 ± 0.09 (p < 0.01) and PFOS to 0.48 ± 0.13, indicating a potent inhibitory effect. These compounds also decreased IgM levels, although the effect was generally less pronounced, especially for PFOA. PFHxS showed significant suppression of both IgM and IgG at 10 μg/mL. In contrast, PFHxA, PFBS and PFBA had no effect on IgG production, with SI values remaining close to control levels, but were able to statistically significant suppressed the IgM release at 10 μg/mL. Notably, PFBA significantly reduced IgM also at 0.001 μg/mL. This suggests that even short-chain PFAS may impact humoral immunity under specific conditions, specifically for IgM production in male donors.

In female donors (see Table 2), the pattern of immunosuppression was generally similar, though less pronounced for IgG and more for IgM for long-chain PFAS. PFNA again reduced IgG and IgM production, with significant effects observed at the highest concentrations. PFOS and PFOA significantly decreased IgM at all concentrations tested. In contrast, short-chain PFAS generally did not significantly affect antibody production in female donors. A similar suppression is visible at the highest concentration of IgM when comparing male donors, but this is not statistically significant due to the deviation.

**FIGURE 1:**
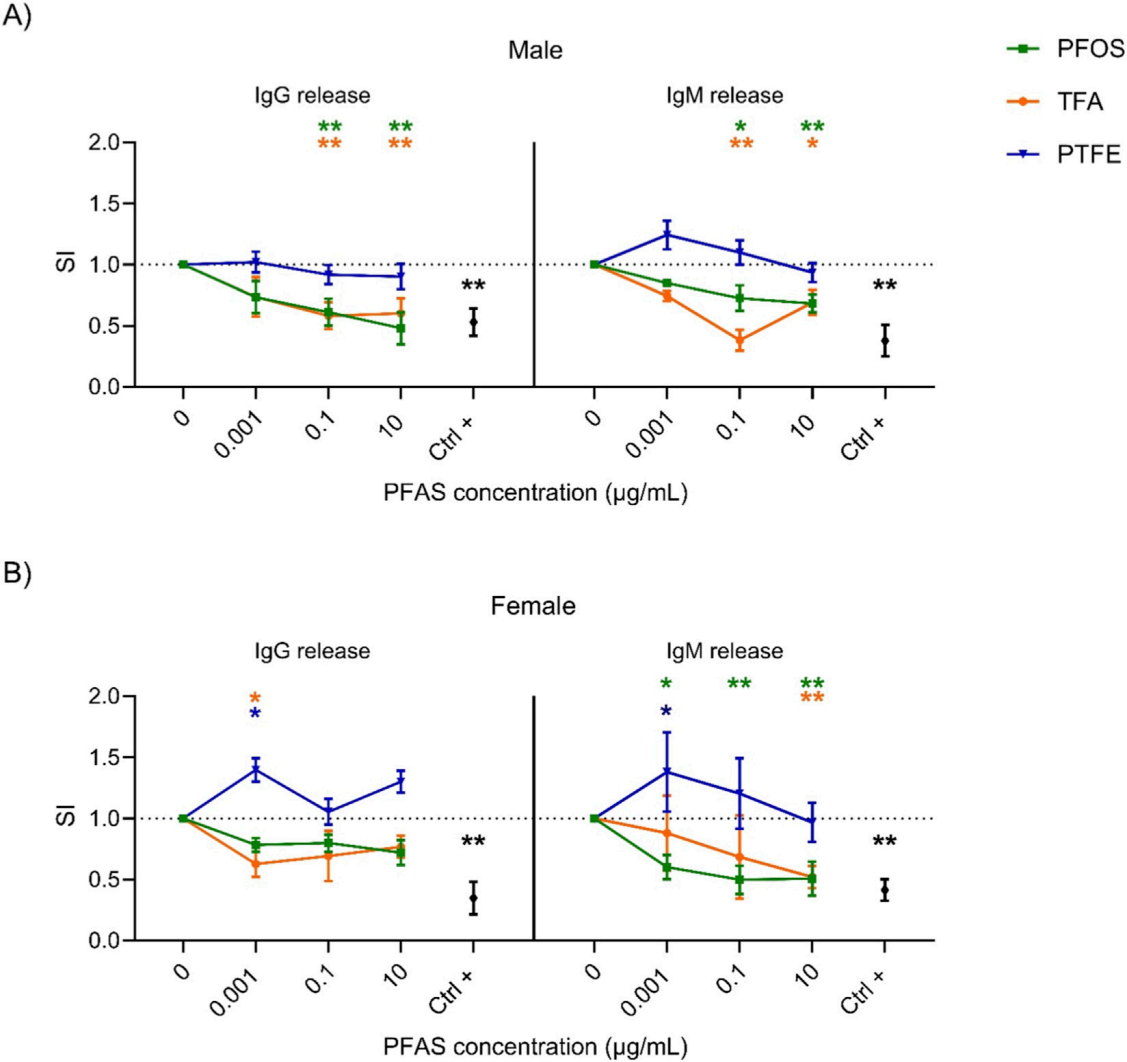
Effect of the PFOS, PTFE and TFA on IgG and IgM release. Analysis of the release of IgM and IgG after PFAS treatment in male **(A)** and female **(B)** human donors. Human PBMC (1.26 × 10^6^ cells/mL) were treated for 24 h with increasing concentration of PFAS (0.001, 0.1 and 10 μg/mL) or with the positive control Rapamycin (Ctrl +, 2 ng/mL). Then, cells were stimulated with 1 μg/mL of ODN2006 and 100 IU/mL of IL-2 to induce the B cell differentiation. After 6 days, the release of IgG and IgM was analyzed. Results are expressed as SI of total IgG and IgM respect to vehicle treated cells. Each value represents the mean ± SEM, with n = 5 different male and five different female donors. Statistical analysis was carried out using two-way ANOVA, followed by Dunnett’s test for PFAS vs. Ctrl (represented by the dot line at 1), while unpaired t-test for Ctrl + vs. Ctrl. Results were considered significant if p ≤ 0.05, with *p ≤ 0.05 and **p ≤ 0.01 vs. Ctrl. The color of the asterisk merges the color of the chemicals.

Having analyzed the effects of both long- and short-chain PFAS, we sought to investigate whether the observed impact on antibody production depended directly on chain length. To this end, we selected two extreme examples: the fluoropolymer PTFE and ultra-short-chain TFA. The cells were then exposed to these two compounds in order to explore potential correlations between chain length and immunotoxicity. Figure 1 shows the results of a comparison of the release of IgG and IgM in response to exposure to the reference compound, PFOS, as well as two compounds representing opposite extremes of chain length: TFA and PTFE. PFOS was selected as the reference compound due to its well-established immunotoxic profile and widespread relevance in regulatory and scientific contexts. The data confirm that PFOS consistently suppresses antibody production in a dose-dependent manner in both male and female donors. This suppression is evident for both IgG and IgM, with statistically significant reductions observed starting from intermediate concentrations (0.1 μg/mL). When the effects of the ultra-short-chain compound TFA are compared to those of PFOS, an unexpected similarity emerges in male donors (1A), TFA induces a comparable reduction in the release of both IgG and IgM, with significant inhibition occurring at higher concentrations. The same trend was also observed for female donors (1B). This suggests that extremely short-chain PFAS can produce immunosuppressive effects similar in magnitude to those observed with long-chain analogues despite their difference in the carbon-chain and presumed lower bioaccumulation potential. Conversely, PTFE exhibited a markedly different pattern. In male donors (1A), exposure to PTFE did not result in significant suppression of antibody production, with SI values remaining close to or slightly above control levels across all concentrations. In female donors (1B), PTFE even elicited a moderate and statistically significant increase in IgG and IgM release at the lowest concentration (0.001 μg/mL), suggesting a potential immunostimulatory effect that is unique to this compound and gender subgroup.

These observations highlight that chain length alone is not a sufficient predictor of immunotoxic potential. While shorter-chain PFAS often exhibits reduced bioactivity, ultra-short TFA can still exert potent suppressive effects. In contrast, PTFE appears to be largely inactive or even stimulatory. Additionally, gender-specific differences emerged, with female donors exhibiting greater variability and, in some cases, opposite trends (e.g., increased antibody release in response to PTFE).

### 3.2 Effects of PFAS on the maturation of DCs

**FIGURE 2:**
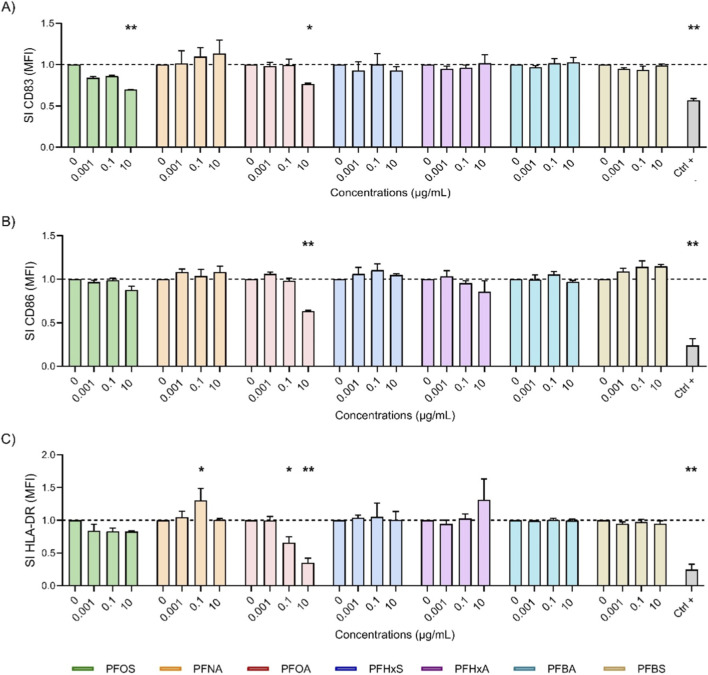
Effect of PFAS on surface marker expression after 24 h of DC maturation. Analysis of the expressions of CD83 **(A)**, CD86 **(B)** and HLA-DR **(C)** were reported. iDCs (1 × 10^6^ cells/mL) were treated with increasing concentration of selected PFAS or the positive control DEX (150 μg/mL, Ctrl +) for 24 h. Then, the maturation cocktail composed of rhIL-4 (3000 IU/mL), rhGM-CSF (1500 IU/mL), rhTNF-α (2000 IU/mL) and ionomycin (200 ng/mL) was added for additional 24 h to acquire the properties of mDCs. After 24 h the surface markers were evaluated. The dashed line was set to 1, in relation to the control cells (mDCs). The results of the maturation are reported and expressed as SI of MFI. Any value shown in the graph represents the mean ± SEM, with n = 3 independent experiments. Statistical analysis was carried out using two-way ANOVA, followed by Dunnett’s test for PFAS vs. mDCs, while unpaired t-test for Ctrl + vs. mDCs. Results were considered significant if p ≤ 0.05, with *p ≤ 0.05 and **p ≤ 0.01vs mDCs.

**FIGURE 3:**
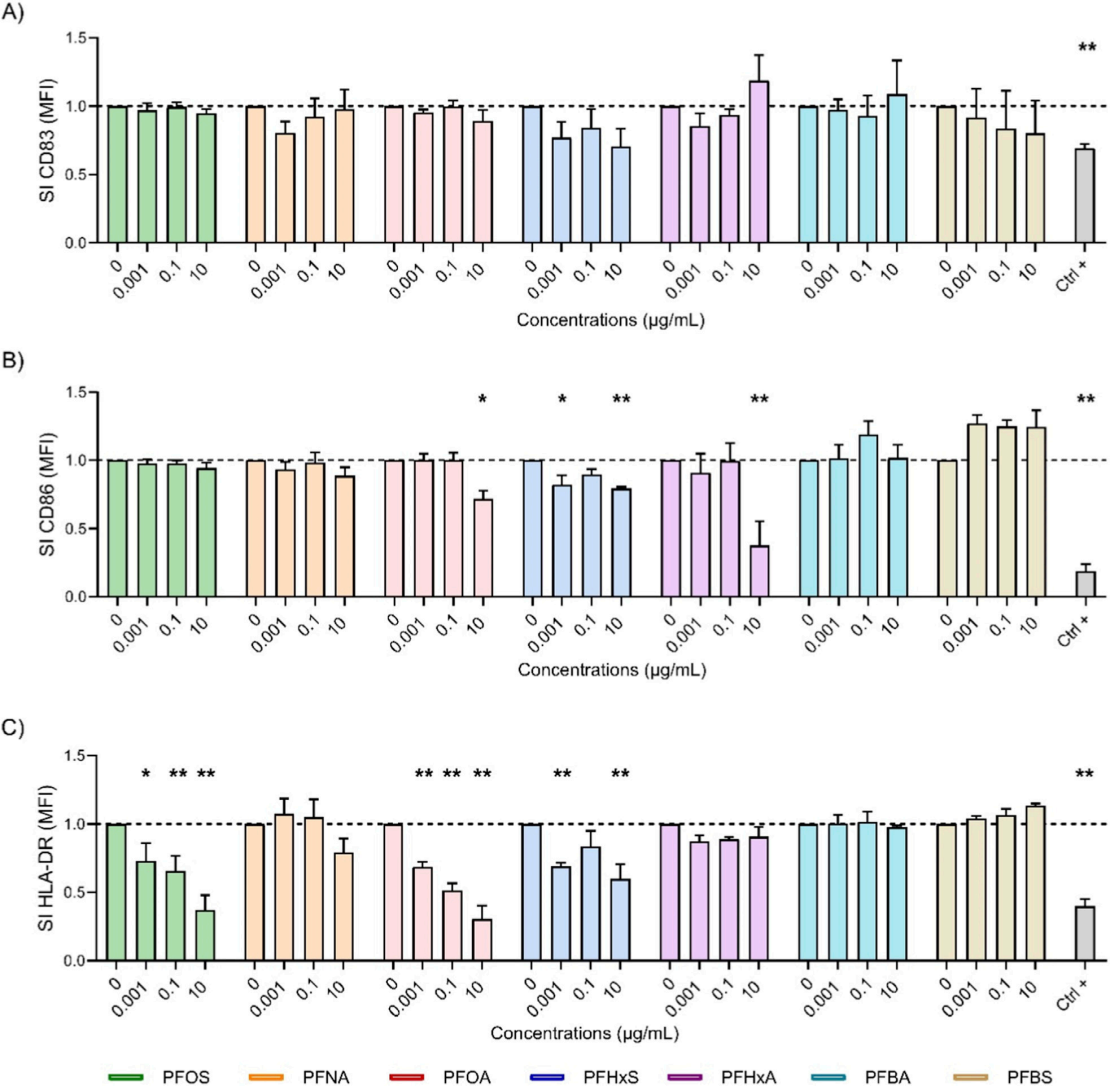
Effect of PFAS on surface marker expression after 72 h of DC maturation. Analysis of the expressions of CD83 **(A)**, CD86 **(B)** and HLA-DR **(C)** were reported. iDCs (1 × 10^6^ cells/mL) were treated with increasing concentration of selected PFAS or the positive control DEX (150 μg/mL, Ctrl +) for 24 h. Then, the maturation cocktail composed of rhIL-4 (3000 IU/mL), rhGM-CSF (1500 IU/mL), rhTNF-α (2000 IU/mL) and ionomycin (200 ng/mL) was added for additional 72 h to acquire the properties of mDCs. After 72 h the surface markers were evaluated. The dashed line was set to 1, in relation to the control cells (mDCs). The results of the maturation are reported and expressed as SI of MFI. Any value shown in the graph represents the mean ± SEM, with n = 3 independent experiments. Statistical analysis was carried out using two-way ANOVA, followed by Dunnett’s test for PFAS vs. mDCs, while unpaired t-test for Ctrl + vs. mDCs. Results were considered significant if p ≤ 0.05, with *p ≤ 0.05 and **p ≤ 0.01vs mDCs.

The recognition and processing of antigens by APCs is essential for triggering specific immune responses, and DCs play a key role in this process. Although primary DCs can be used, they are difficult to isolate, and the results vary. Therefore, differentiated THP-1 cells were chosen as a more reliable alternative. The methods of Berges et al. (2005) provides a reproducible protocol for generating DC-like cells from THP-1 monocytes. The THP-1 cells were first differentiated into iDCs using rhIL-4 and rhGM-CSF and then exposed to PFAS for 24 h. A maturation cocktail containing rhIL-4, rhGM-CSF, rhTNF-α and ionomycin was then added to induce mDCs. Maturation was assessed at 24 and 72 h by measuring the surface markers CD83, CD86 and HLA-DR. CD83 is an activation marker that promotes the upregulation of MHC II and CD86, which are crucial for T cell activation. CD86 provides the co-stimulatory signals that are necessary for T cell adhesion and activation, interacting with CD28 to stimulate cytokine production and T cell proliferation and differentiation. HLA-DR, which is part of MHC II, presents antigens to T helper (Th) cells and regulates T cell responses and antibody production (Li et al., 2019; Kaiko et al., 2008). Differentiation into iDCs was confirmed after 5 days by increased expression of CD40, CD80 and CD86, as measured by flow cytometry and quantified by fluorescence intensity relative to controls (data not shown). The impact of the selected PFAS on mDCs was evaluated by treating iDCs with increasing concentrations of PFOS, PFOA, PFNA, PFHxS, PFHxA, PFBS, PFBA, or DEX as a positive control, for 24 h. A maturation cocktail was then added to each treatment condition to induce mDCs. The cells were analyzed 24 and 72 h after the maturation cocktail was added, with the expression of the surface markers CD83, CD86 and HLA-DR being assessed via flow cytometry. The results are presented in Figures 2, 3.

Figure 2 illustrates the impact of various PFAS on the expression of DCs maturation markers (CD83, CD86 and HLA-DR) after 24 h of exposure. iDCs were treated with increasing concentrations (0.001, 0.1 and 10 μg/mL) of the selected PFAS or DEX (Ctrl +), and the expression of the markers was quantified as a SI of MFI, normalized to the expression of the markers in mDCs controls (dashed line). PFOA (pink) significantly suppressed all three markers, notably decreasing HLA-DR (2C) even at low concentrations. It also significantly reduced CD86 (2B) and CD83 (2A) at 10 μg/mL, further supporting its immunosuppressive potential. Notably, PFOS reduced CD83 even at low doses, indicating potent inhibition of DCs maturation. By contrast, PFHxS (blue), PFHxA (purple), PFBA (cyan) and PFBS (gold), did not significantly affect marker expression at any tested concentration. Their profiles were similar to those of untreated mDCs, suggesting a minimal impact on DCs maturation. These results suggest that the inhibitory effects on DCs maturation are not uniformly associated with chain length. Rather, among the compounds tested, PFOA at the highest concentration showed the most pronounced reduction in HLA-DR, CD83, and CD86 expression, whereas other PFAS, including short-chain analogues, appeared to be less disruptive after 24 h of maturation.

Figure 3 shows the impact of various PFAS on the expression of surface markers after 72 h of DCs maturation. Long-chain PFAS, notably PFOS (green) and PFOA (pink), exhibited potent inhibitory properties. PFOS caused a consistent and significant reduction in HLA-DR (3C) across all concentrations, with the suppression of CD83 at 24 h being largely reversed by 72 h (2A). PFOA exhibited a suppression of CD86 at the highest concentration (3B) and a pronounced dose-dependent decrease in HLA-DR (3C), including significant reductions in HLA-DR at lower concentrations. The impact, measured as the stimulation index (SI), was greater at 72 h than at 24 h (lower SI values), indicating that PFOA has an enhanced suppressive effect over time. PFHxS (blue) also showed a statistically significant reduction in both CD86 and HLA-DR at lower and higher dose. This suggests that there is immunomodulatory activity with prolonged exposure also visible for CD83 (3A) although not statistically significant. In contrast, the other short-chain PFAS, PFBA (cyan) and PFBS (gold), generally did not significantly alter the expression of CD83, CD86 or HLA-DR, with SI values close to those of the controls. This indicates that there was a minimal impact on DCs maturation after 72 h. An exception was observed for PFHxA (purple), which affected CD86 expression more prominently than any other PFAS tested. This was in line with the suppression induced by the positive control, DEX. Notably, PFBS at 10 μg/mL exhibited an increase in CD86 expression. Overall, these results confirm that long-chain PFAS, especially PFOA and PFOS, sustained immunosuppressive effects on key DCs maturation markers. In contrast, short-chain PFAS display negligible influence, which reinforces their comparatively lower immunotoxic risk. This emphasizes the pivotal role of chain length in PFAS immunotoxicity and risk assessment.

Overall, these results demonstrate that both the shortest and longest PFAS can produce distinct and sometimes unexpected immunological outcomes, emphasizing the need for compound-specific evaluations rather than making assumptions based on carbon chain length alone.

## 4 Discussion

Our study provides new insights into the immunotoxicity of PFAS by directly comparing fluoropolymer, long-, short- and ultra-short chain representatives using human-relevant NAMs. The results obtained aligned with a growing body of evidence from *in vivo* animal studies and human epidemiology and reinforce the importance of integrating NAMs into immunotoxicology risk assessments.

With regard to DC maturation, our data revealed effects that were dependent on both chain length and time. Long-chain PFAS such as PFOA and PFOS showed clear inhibitory effects on maturation markers, whereas short-chain PFAS were largely inactive at early time points. At 72h, the suppression of HLA-DR by PFOS and PFOA became more pronounced and persistent, while effects on CD83 and CD86 appeared more transient or compound-specific. PFHxS and PFHxA displayed modest activity only upon prolonged exposure. Taken together, these observations highlight that the kinetics of DC maturation markers are not uniform. HLA-DR showed the most consistent and progressive inhibition, suggesting interference with sustained antigen presentation pathways and MHC class II regulation. In contrast, CD83 and CD86 are more rapidly inducible and appear to be affected more transiently, with partial recovery over time. This differential pattern emphasises the importance of assessing multiple markers and time points to capture the various aspects of DC dysfunction. In our models, the suppression of DC maturation by long-chain PFAS was accompanied by significant inhibitory effects on antibody production in PBMCs. These findings agree with previous animal studies demonstrating that PFOS and PFOA reduce antibody responses in rodents, often at doses relevant to human exposure (Peden-Adams et al., 2008; Dong et al., 2009; DeWitt et al., 2012). For instance, exposure to PFOS in mice results in substantial reductions in IgM and IgG production following immunization and impairs TDAR (Peden-Adams et al., 2008; DeWitt et al., 2009). Similarly, PFOA has been shown to suppress splenic antibody production and alter cytokine profiles in rodent models (DeWitt et al., 2012). These findings in animals are reflected in human epidemiological studies, in which higher blood levels of PFOS and PFOA have been associated with reduced vaccine efficacy and an increased risk of infection in children (Grandjean et al., 2012; Dalsager et al., 2016; Abraham et al., 2020). Together with *in vitro* data published by Corsini et al. and Iulini et al. (2025), our results confirm that PFOS is an appropriate reference compound for long-chain PFAS as it produces consistent, robust immunosuppressive effects across both humoral and cell-mediated endpoints.

Among the long and short-chain PFAS tested, PFNA, PFOS and PFOA exhibited significant inhibitory effects on immunoglobulin release, even at low concentrations. By contrast, PFHxS, PFHxA, PFBA and PFBS had only modest effects, which were generally only evident at the highest concentrations tested. At the starting point, this difference appears to be primarily related to their differential cellular uptake as demonstrated by Iulini et al. (2025) –article under revision). Using the *in vitro* distribution model developed by Armitage et al. (2021), the intracellular mass fraction for each compound was estimated. The results showed that long-chain PFAS accumulated within cells more than short-chain PFAS. For example, PFOS accumulated to ∼25% of total mass, whereas PFBS accumulated to <1% (Iulini et al., 2025). A strong inverse correlation emerged between intracellular availability and immunoglobulin production: the higher the intracellular fraction, the more pronounced the immunosuppressive effect. In contrast, protein binding and lipid partitioning did not correlate with biological activity (Iulini et al., 2025). Accordingly with those results, the lower biological activity observed for short-chain PFAS is likely due to their limited cellular distribution, which restricts effective intracellular concentrations despite their relatively lower protein binding. This mechanistic insight could explain why short-chain PFAS are perceived as having a comparatively favourable, though not risk-free, toxicological profile compared to their long-chain analogues, particularly under chronic exposure.

Interestingly, while short-chain PFAS generally showed minimal immunosuppressive effects in male donors, female donors exhibited a non-significant trend toward decreased antibody levels, indicating possible gender-dependent sensitivity. Notably, the ultra-short-chain compound TFA produced immunosuppressive effects comparable to PFOS, challenging the assumption that shorter chain length equates to lower immunotoxicity and departing from the above-observed hypothesis. This challenges the prevailing assumption that shorter chain length necessarily correlates with lower immunotoxic potential. This suggests that the potent antibody suppression observed with TFA is unlikely to be solely explained by cellular accumulation or bioavailability, as its intracellular presence is relatively low and similar to other short-chain compounds. However, the notably low molecular weight of TFA (∼114 g/mol) means that at an equivalent mass concentration (e.g., 10 μg/mL), its molar concentration (∼88 µM) is more than four times higher than that of PFOS (∼20 μM at 10 μg/mL, molecular weight ∼500 g/mol). At the same mass concentration, the corresponding molar values were ∼47 µM for PFBA, ∼32 µM for PFHxA, ∼33 µM for PFBS, ∼25 µM for PFHxS, ∼24 µM for PFOA, and ∼22 µM for PFNA (value also reported in Supplementary Material), indicating that although short-chain PFAS generally yield higher molar concentrations than long-chain analogues, the overall range remains comparable across compounds, except for TFA. This higher molar abundance could contribute to the pronounced immunosuppressive effects observed. Nevertheless, a plausible explanation for TFA’s pronounced immunosuppressive activity is that it operates via a distinct mechanism of action compared to both long- and typical short-chain PFAS, beyond differences in cellular uptake or molar concentration. Reporting these values in µM facilitates comparison with other *in vitro* studies and supports a more consistent interpretation of the observed effects.

It is also important to highlight that only a few immunotoxicological assessments have been conducted for TFA. These have mostly involved immunophenotyping of F1 rats in the OECD TG 443 study, as well as white blood cell and lymphoid organ analyses in adult animals (ECHA dossier, 2025). However, the lack of a dedicated immunotoxicity cohort has hindered detailed evaluation. Observed effects included a reduction in the number of splenic T, B, NK and CD4^+^ and CD8^+^ cells, as well as monocytes and neutrophils, in both sexes at all doses. A decreased percentage of CD4^+^ and an increased percentage of CD8^+^ T cells were observed at the lowest dose in females, with no other significant immunophenotyping changes reported. In view of the limited immunotoxicity data for TFA and the results obtained in the present study, it is crucial from a regulatory perspective to address this gap by implementing dedicated immunotoxicity testing strategies, including, where feasible, the use of NAMs specifically targeting immune endpoints, especially considering that TFA is both a PFAS and a degradation product of longer-chain PFAS.

The immunotoxicity of long-chain PFAS such as PFOS and PFOA is primarily linked to activation of nuclear receptors like PPAR-α, which modulates lipid metabolism and immune cell differentiation, leading to suppression of B- and T-cell functions (DeWitt et al., 2012; NTP, 2016; Schrenk et al., 2020). Additionally, these compounds inhibit NF-κB signaling, leading to suppression of genes involved in innate and adaptive immunity (Ehrlich et al., 2023). Short-chain PFAS, on the other hand, tend to have weaker affinity for PPAR-α and exhibit less pronounced effects on these classical nuclear receptor pathways (Gomis et al., 2018; Amstutz et al., 2024). Their immunotoxicity is often associated with mitochondrial dysfunction and oxidative stress pathways that impair immune cell energy metabolism and viability (Liu et al., 2023; Obiako et al., 2024). However, these mechanisms generally produce milder immunosuppressive effects, consistent with their lower bioaccumulation and potency. Transcriptomic studies reveal that PFAS exposure downregulates genes involved in cholesterol biosynthesis and clearance (notably for long-chain PFAS), upregulates lipolysis-related genes (notably for short-chain PFAS), and broadly suppresses gene sets governing both innate and adaptive immunity, with sulfonate-type PFAS exerting particularly strong effects (Reardon et al., 2021; Addicks et al., 2025). Conversely, as expected for a high-molecular-weight polymer, PTFE did not suppress antibody production and, curiously, was associated with increased antibody release in female donors, displayed a distinct response compared to perfluoroalkyl acids. While this effect requires further confirmation, it raises the possibility of immunostimulatory properties that have not been previously described and should be further investigated.

Our use of NAMs, specifically human PBMCs antibody production assays and THP-1-derived DCs maturation models, demonstrates their utility for high-content, human-relevant immunotoxicity screening of PFAS. This assessment closely aligns with regulatory risk assessment priorities (EPA, 2016; Schrenk et al., 2020). Notably, impaired DCs maturation in the THP-1 assay is an early indicator of reduced antigen presentation capacity, which is a crucial upstream event in the activation of adaptive immune responses, including T cell priming and B cell help. Conversely, the direct suppression of IgM and IgG antibody release in PBMCs cultures reflects the impact of PFAS on the effector phase of humoral immunity, which is mechanistically linked to the decreased vaccine responsiveness observed in epidemiological studies (Grandjean et al., 2012; Dalsager et al., 2016; Abraham et al., 2020). Using both assays simultaneously thus reveals whether PFAS primarily disrupts adaptive responses via APCs, potentially leading to broader immune deficits, or exert more immediate inhibitory effects on antibody-producing B cells. The observation of adverse effects in both models suggests that multiple nodes of the adaptive immune system are compromised, which can amplify immune suppression, resulting in greater clinical risk, including decreased vaccine efficacy and increased susceptibility to infectious and allergic diseases. Therefore, this integrative NAMs-based approach offers an important screening tool for regulatory immunotoxicity, as well as providing scientific insight into which compartment of the adaptive immune system is most vulnerable to PFAS disruption. It is important also to highlight that NAMs overcome many limitations of animal models, such as differences in immune system development and chemical kinetics between species (Fenton et al., 2021). Recent advances, including 3D lymphoid models and co-culture systems, further enhance the physiological relevance of *in vitro* assays. Integrating them with PBK modelling can also bridge the gap between *in vitro* concentrations and human exposure, thus improving extrapolation to real-world risk (Corsini et al., 2024). Despite these advances, several limitations remain. Our study focused on acute single-compound exposures, which do not fully reflect the chronic, low-dose, and mixture scenarios that characterize real-world PFAS exposure. Combined exposures to multiple PFAS and their possible additive or synergistic immunotoxic effects were not evaluated here and should be a priority for future research (Maddalon et al., 2023). Furthermore, although PFAS are generally considered resistant to biotransformation, our *in vitro* models do not account for metabolic activation, detoxification or reabsorption. These factors may significantly influence immunotoxic potential *in vivo* (Fenton et al., 2021). Moreover, our findings underscore the need for compound-specific assessment rather than broad generalizations based solely on carbon chain length (Gomis et al., 2018; Amstutz et al., 2024). However, a key limitation in achieving this goal has been the development of PBK models capable of accurately predicting the ADME (absorption, distribution, metabolism, and excretion) of PFAS, which, as demonstrated also in this article, exhibit distinct distribution, and consequently kinetics, and effects depending on their structure. A preliminary attempt to address this challenge has been presented by Iulini et al. (2024a), but further refinement and implementation of such models will be essential as more compound-specific properties of individual PFAS in humans are elucidated. Integrating NAMs data with PBK modeling offers promising avenues for translating *in vitro* observations to human-relevant exposure levels. For example, PBK models have been successfully applied to estimate PFOS and PFOA concentrations in serum following dietary intake and to compare these internal doses with *in vitro* effect concentrations (Schrenk et al., 2020; Corsini et al., 2024; Iulini et al., 2024b). Coupling NAM-derived potency data with PBK simulations and human biomonitoring data will be critical to improve the predictive value of *in vitro* assays and inform regulatory decision-making.

## 5 Conclusion

In summary, the results obtained reinforce the higher immunotoxic potential of long-chain PFAS, the relative safety of most short-chain alternatives, and the unexpected risk posed by the ultra-short-chain compounds TFA. The use of NAMs has proven effective in detecting and characterizing these effects, thus supporting their broader adoption in regulatory science. Continued refinement of NAMs, combined with mechanistic, epidemiological and modelling approaches, will be essential to fully characterize and mitigate the immunological risks of PFAS.

## Data Availability

The raw data supporting the conclusions of this article will be made available by the authors, without undue reservation.
