# Supplementary material for: Decoding PFAS immunotoxicity: a NAMs-based comparison of short vs. long chains
Source: Front Toxicol. 2025 Nov 19;7:1665163. doi: 10.3389/ftox.2025.1665163 (PMC12672224; doi:10.3389/ftox.2025.1665163)
Supplement: Supplementary file 1 [file Table1.docx]

Supplementary Material

Decoding PFAS immunotoxicity: a NAMs-based comparison of short vs long chains

Martina Iulini^1,*^, Valentina Galbiati^1^, Marina Marinovich^1^ and Emanuela Corsini ^1^

^1^Laboratory of toxicology and risk assessment, Department of Pharmacological and Biomolecular Sciences “Rodolfo Paoletti”, University of Milan, Milan, Italy

*** Correspondence:**Martina Iulini
martina.iulini@unimi.it

Keywords: new approach methodologies (NAMs), PFAS, hazard identification, human PBMCs, dendritic cells, in vitro

Table 1S: Comparison of compound concentrations expressed in µg/mL and µM, calculated based on the respective molecular weight.

| **Compound** | **CAS No.** | **MW (g/mol)** | **0.001 µg/mL (µM)** | **0.1 µg/mL (µM)** | **10 µg/mL (µM)** |
| --- | --- | --- | --- | --- | --- |
| PTFE | 9002-84-0 | n.a. | n.a. | n.a. | n.a. |
| PFNA | 375-95-1 | 464.07 | 0.002 | 0.22 | 21.55 |
| PFOS | 1763-23-1 | 500.13 | 0.002 | 0.20 | 20.00 |
| PFOA | 335-67-1 | 414.07 | 0.002 | 0.24 | 24.15 |
| PFHxS | 3871-99-6 | 400.11 | 0.003 | 0.25 | 25.00 |
| PFHxA | 307-24-4 | 314.04 | 0.003 | 0.32 | 31.85 |
| PFBS | 375-73-5 | 300.10 | 0.003 | 0.33 | 33.32 |
| PFBA | 375-22-4 | 214.04 | 0.005 | 0.47 | 46.74 |
| TFA | 76-05-1 | 114.02 | 0.009 | 0.88 | 87.70 |
